# A Tailored Gender-Sensitive mHealth Weight Loss Intervention (I-GENDO): Development and Process Evaluation

**DOI:** 10.2196/38480

**Published:** 2022-10-27

**Authors:** Magdalena Pape, Tanja Färber, Caroline Seiferth, Tanja Roth, Stefanie Schroeder, Joerg Wolstein, Stephan Herpertz, Sabine Steins-Loeber

**Affiliations:** 1 Department of Psychosomatic Medicine and Psychotherapy LWL-University Hospital of the Ruhr-University Bochum Bochum Germany; 2 Department of Clinical Psychology and Psychotherapy University of Bamberg Bamberg Germany; 3 Department of Pathopsychology University of Bamberg Bamberg Germany

**Keywords:** mobile health, mHealth, eHealth, tailoring, gender, weight loss intervention, mobile phone

## Abstract

**Background:**

Given the increase in the prevalence of overweight and obesity worldwide, the number of digital weight loss interventions has also risen. However, these interventions often lack theoretical background and data on long-term effectiveness. The consideration of individual and gender differences in weight-related psychological parameters might enhance the efficacy and sustainability of mobile-based weight loss interventions.

**Objective:**

This paper presented an introduction to and the process evaluation of a 12-week gender-sensitive mobile health (mHealth) weight loss intervention (I-GENDO) combining computer-based and self-tailoring features.

**Methods:**

Between August 2020 and August 2021, individuals with overweight (BMI 25.0-29.9 kg/m²), those with obesity class I (BMI 30.0-34.9 kg/m²), and those with obesity class II (BMI 35.0-39.9 kg/m²) were recruited to the I-GENDO project, a multicenter study in Germany. The mHealth intervention aimed at targeting individual psychological factors associated with the development and persistence of overweight and obesity (eg, emotional eating) using computer-based tailoring. Moreover, the intervention took a gender-sensitive approach by implementing self-tailoring of gender-targeted module versions. The computer-based assignment of the main modules, self-selection of gender-targeted module versions, and use patterns were evaluated while considering gender. Moreover, gender differences in the usability assessment were analyzed.

**Results:**

Data from the intervention arm of the study were processed. A total of 116 individuals with overweight and obesity (77/116, 66.4% women; age mean 47.28, SD 11.66 years; BMI mean 33.58, SD 3.79 kg/m^2^) were included in the analyses. Overall, the compliance (90/109, 82.6%) and satisfaction with the app (mean 86% approval) were high and comparable with those of other mobile weight loss interventions. The usability of the intervention was rated with 71% (5.0/7.0 points) satisfaction. More women obtained the main module that focused on emotion regulation skills. Most men and women selected women-targeted versions of the main modules. Women used the app more frequently and longer than men. However, women and men did not differ in the progress of use patterns throughout the intervention.

**Conclusions:**

We developed a tailored gender-sensitive mHealth weight loss intervention. The usability of and engagement with the intervention were satisfactory, and the overall satisfaction with the intervention was also high. Gender differences must be considered in the evaluation of the effectiveness and sustainability of the intervention.

## Introduction

Within the last few decades, a vast number of digital health apps have been developed worldwide [1,2]. eHealth interventions (ie, mobile health [mHealth] interventions) are cost-effective and feasible in everyday life and represent a useful addition to analog health care services, not only in times of a worldwide pandemic [3]. In 2021, 87% of German adults and adolescents aged >14 years owned a smartphone, and 27% reported using mHealth interventions regularly [4]. The use of mHealth interventions requires an active and self-determined engagement of the user and therefore facilitates behavioral changes [5]. For example, mHealth lifestyle interventions show good efficacy in promoting healthy behaviors such as dietary intake and physical activity [6-10]. Therefore, they are promising tools that could promote behavioral change in participants wishing to reduce weight [11]. However, most available interventions to date demonstrate only short-term effects of behavioral change, whereas long-term effectiveness, especially regarding weight loss, has either not been investigated or not been demonstrated [12-14]. An explanation for the lack of effects is that most weight loss apps have not been developed from a scientific background and thus lack sufficient consideration of psychological evidence-based strategies [15], which are an important aspect of effective weight loss programs (WLPs) according to international guidelines [16,17]. Moreover, most weight loss apps have been developed on a *one-size-fits-all* approach, despite indications from prior studies that targeted (tailored) interventions are more effective [18-20].

The term “tailoring” refers to the customization of a feature of an intervention based on the individual characteristics of the participants [21]. The participants might customize an intervention based on their own preferences (self-tailoring), or they might receive individualized interventions in which the program tailors the content, usually based on algorithms (computer-based tailoring). In the latter case, tailoring can be based on data from 1 assessment (static tailoring) or adapted to different assessments within an intervention process (dynamic tailoring). Studies have indicated that participants feel more strongly addressed by individualized interventions, are more satisfied with them, and are subsequently more engaged in their use, which enhances the efficacy of the programs [6,11,19,22-25]. Various psychological aspects are involved in the development and maintenance of overweight and obesity, including the experience of weight-related stigmatization [26], maladaptive coping strategies [27], or dysfunctional eating behaviors [28]. Therefore, developing computer-based tailoring features that consider such psychological aspects might be a key element in the optimization of digital WLPs.

Gender differences in the development and treatment of obesity and overweight have also been investigated [29,30]. In Germany, more men (43.3%) develop overweight (BMI 25-29.9 kg/m²) compared with women (28.8%), but there are no gender differences in the prevalence of obesity (BMI >30.0 kg/m²), with increasing prevalence rates in the past decades among both genders [31,32]. Men with overweight and obesity are less likely to accurately perceive their weight and are less dissatisfied with their overweight status [29]. Moreover, gender differences in physical activity, eating behavior, and weight-related psychological parameters have been reported. For example, women engage more often in problematic eating behaviors, such as emotional eating (EE) and craving of special foods than do men [33]. Women consistently report higher levels of perceived stress and engage more in emotion-focused coping, such as rumination, whereas men often use problem-focused or avoidant coping strategies [34,35]. On average, men are more physically active [36]. Some biological sex differences have been published; for instance, in males, fat depositions are often in the visceral depot, which increases their risk for cardiovascular disorders [37-39]. More women participate in WLPs, yet the participating men lose more absolute weight [40,41]. Results on the adherence to WLPs are heterogeneous, depending on the intervention type, among other factors [42-44]. On the basis of reviewed studies, investigating the effect of gender on overweight and obesity outcomes to improve the effectiveness of WLPs is an important research agenda. A recently published meta-analysis comparing the effects of gender-targeted and gender-neutral WLPs however revealed no differences in weight-related outcomes, although gender-targeted interventions were more effective in promoting activity and improving nutrition [45]. However, the included *gender-targeted* WLPs were offered either to male or to female participants based on sex. We support the idea that psychological interventions should be gender sensitive instead of gender dichotomous and assume an increase in the effectiveness of the intervention if it is gender sensitive [46]. Therefore, to avoid prejudiced gender-based distinctions between individuals with overweight and obesity, we recommend implementing gender-sensitive self-tailoring features.

Against this background, we aimed at developing a smartphone-based psychological and gender sensitive weight-loss intervention with computer-based and self-tailoring features. In the first part of this paper, we have described the development process of the app with particular focus on the tailoring features of the intervention. The subsequent process evaluation focuses on the evaluation of the app with regard to the psychological and gender-sensitive tailoring features, use patterns, and satisfaction with the app derived from a sample of 116 participants taking part in the I-GENDO project [47].

## Methods

### The I-GENDO Project

The project “Gender-sensitive enhancement of common weight-loss strategies for overweight and obesity: A personalized smartphone app” was proposed by the University of Bamberg, Departments of Clinical Psychology and Psychotherapy and Pathopsychology, in cooperation with LWL-University Hospital of Ruhr-University Bochum, Department of Psychosomatic Medicine and Psychotherapy, and funded by the Federal Ministry of Education and Research of Germany (01GL1719A/B). The project was preregistered (ClinicalTrials.gov identifier: NCT04080193).

### Ethical Considerations

This study was conducted in accordance with the Declaration of Helsinki. The Institutional Review Board of Ruhr-University Bochum approved the study (number 18-6415). All participants were informed about the study and provided written informed consent.

### Development of the mHealth Intervention I-GENDO

From September 2017 to November 2019, a modular app system was developed at the University of Bamberg in cooperation with an external software provider (groupXS Solutions GmbH).

**Figure 1:**
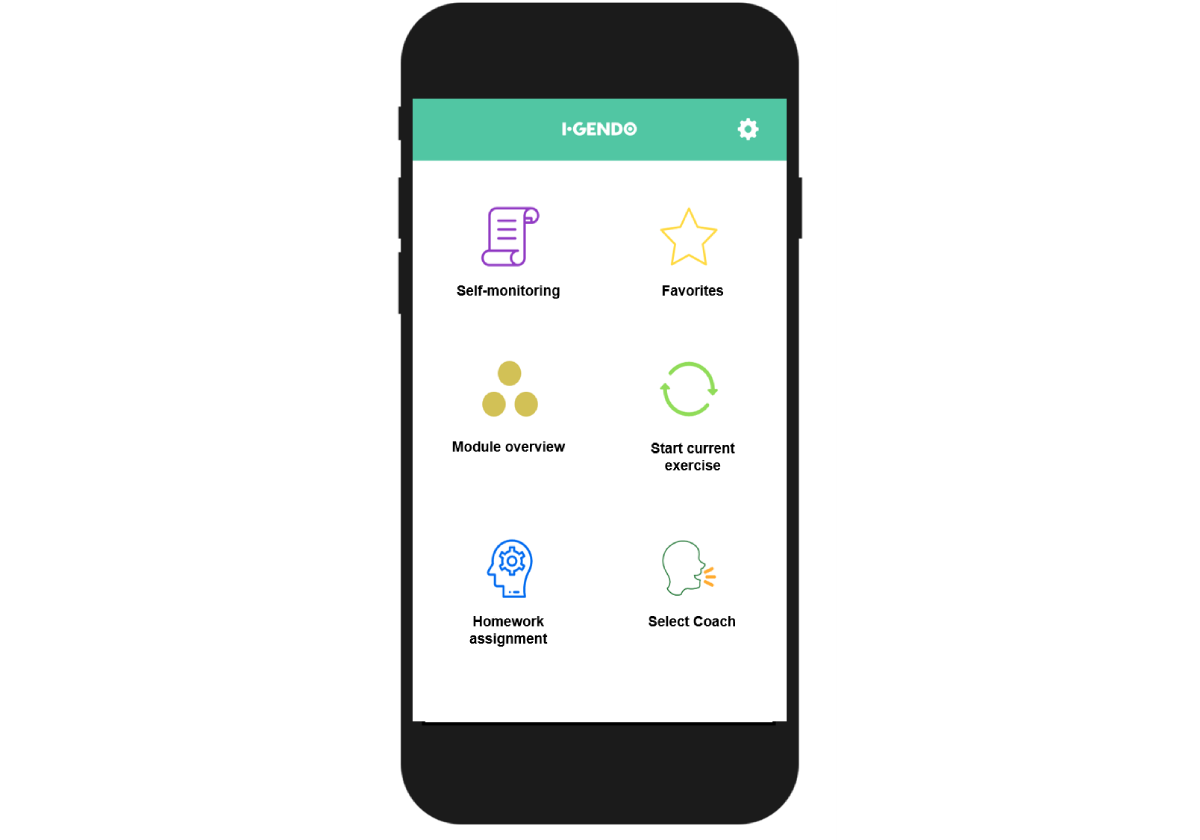
The I-GENDO app interface.

Figure 1 provides an overview of the I-GENDO app interface. The app provided the following elements: module-based psychological intervention; selection of an accompanying coach; and self-monitoring of hunger, appetite, and mood.

The content of the modules was based on the existing evidence-based manuals, qualitative data from focus groups of individuals with overweight and obesity, and interviews with experts in the field of psychological treatment of obesity. To implement a gender-sensitive approach, extensive literature reviews were conducted on the disparities between genders in the psychological and behavioral aspects of obesity treatment. Furthermore, a steering committee consisting of experts in the field of prevention and treatment of overweight and obesity, digital transformation, and qualitative data analyses was formed. All principal decisions regarding app development were made in consensus with the members of the steering committee.

On the basis of this information, 7 modules that served as the heart of the 12-week I-GENDO intervention were constructed. Of the 7 modules, 2 modules addressed the introduction to (goal setting) and conclusion (relapse prevention strategies) of the intervention. The remaining 5 modules (main modules) focused on different psychological parameters associated with the development and maintenance of overweight and obesity: stress management skills (*stress module*), emotion regulation skills (*emotion module*), dealing with the consequences of overweight (*consequences module*), self-regulation skills (*control module*), and self-efficacy (*self-efficacy module*). Each module contained 9 sessions, which included psychoeducational elements delivered through texts and videos, several therapeutic tools from different therapeutic approaches (ie, cognitive behavioral therapy, dialectical behavioral therapy, and mindfulness), and various behavior change techniques [48]. These sessions could be repeated as many times as desired, and users could set a short link to their favorite exercises via the toolbox.

Each module was presented in either a women-targeted version (*version A*) or a men-targeted version (*version B*), which differed in terms of knowledge transfer, communication style, and prioritization of topics. For example, in the *stress module*, this was achieved using appealing case examples in the women-targeted version and fact presenting in the men-targeted version to transfer general knowledge about stress. Another example is that the men-targeted version in the *emotion module* highlighted and trained the recognition and labeling of emotions, whereas in the women-targeted version, the association between dysfunctional beliefs and eating behavior was prioritized. Multimedia Appendix 1 [48-77] provides an overview of the operationalization of the gender-sensitive modules and the origin of evidence. The versions were briefly introduced, with both introductions presented on 1 screen page. Participants could then freely choose between *version A* or *B* regardless of biological sex (gender-sensitive instead of gender dichotomous tailoring). Participants were blind to the manipulation of the gender-targeted versions.

### Process Evaluation of the mHealth Intervention I-GENDO

From December 2019 to December 2021, the effectiveness of the 12-week I-GENDO intervention was evaluated in a randomized controlled trial conducted at the University of Bamberg and LWL-University Hospital Bochum, Department of Psychosomatic Medicine and Psychotherapy (ClinicalTrials.gov Identifier: NCT04080193). The main results of the randomized controlled trial will be published elsewhere. In this manuscript, the relevant process evaluation data from the intervention arm were analyzed.

#### Study Sample

Individuals were informed about the I-GENDO project via newspaper articles, radio features, and oral presentations at rehabilitation centers. Participants interested in the study were screened for eligibility (Textbox 1) and, if eligible, were invited to participate. According to the guidelines of the German Association for the Study of Obesity and the German Society for General and Visceral Surgery, individuals with obesity class III (BMI >39.9 kg/m^2^) experience a complex multifactorial framework of severe social, mental, and physical problems and are recommended to undergo bariatric surgery. Therefore, we excluded individuals with obesity class III from participation but provided further support. Because the effect of bariatric surgery on weight loss is mainly driven by physical limitations and varies significantly between the types of operative procedure [78], we decided to exclude individuals who underwent or planned to undergo bariatric surgery. The total study sample consisted of 213 individuals with overweight and obesity, of which 116 (n=77, 66.4% women) were randomly assigned to the intervention group for this study and subsequently included in this analysis.

**Eligibility criteria of the I-GENDO project.**

**Inclusion criteria**

- Legal age (≥18 years)
- Obesity class I or II with subjectively experienced weight-related impairment and a current intention to lose weight
- Overweight (ie, BMI between 25 and 29.9 kg/m²) with weight-related health problems, visceral adipose tissue, or high psychosocial weight-related distress and a current intention to lose weight

**Exclusion criteria**

- Obesity class III (ie, BMI >39.9 kg/m²)
- Current (or within the last 12 months) involvement in a structured weight loss intervention
- Insulin-dependent type 1 diabetes
- Previous or intended bariatric surgery
- Current psychotherapeutic treatment of weight-related health problems
- Weight-enhancing drugs
- Drugs that promote weight loss (eg, antiobesity drugs)
- Weight-enhancing health problems that are not yet treated
- Cancerous disease within the last 5 years
- Current substance-use disorders, major depression, psychosis, suicidal tendency, or pregnancy
- Severe cognitive impairments
- Insufficient knowledge of the German language
- Binge-eating disorder or bulimia nervosa

#### Intervention Phase

Participants in the intervention group received a 12-week tailored app intervention. In the first week of intervention, the introduction module was unlocked for each participant, followed by 9 weeks of tailored intervention comprising 3 of the 5 main modules. Each session of the 3 main modules was unlocked successively between weeks 2 and 9. The basic, minimal content of the remaining 2 modules was provided in the form of mini modules, which were unlocked in week 11. Finally, the conclusion module was provided to each participant in week 12.

#### Tailoring

**Figure 2:**
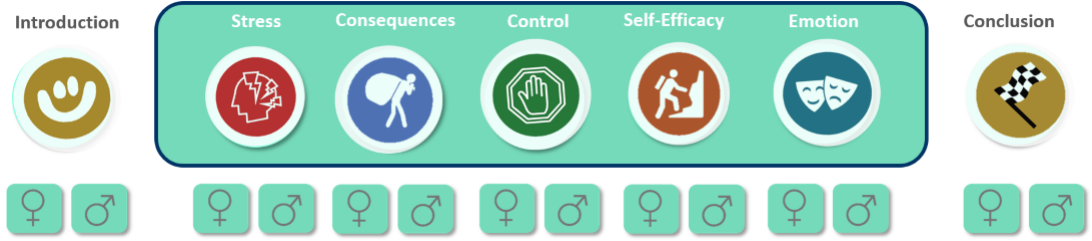
Tailoring features of the I-GENDO intervention. Of the 5 main modules (in the box), 3 were assigned to the participants based on the results of the revised illness perception questionnaire (computer-based tailoring). Each of the modules was presented in either a women- or men-targeted version (self-tailoring).

Figure 2 displays computer-based and self-tailoring features of the intervention. The introduction and conclusion module were mandatory elements framing the intervention that conveyed general content, whereas the main modules targeted individual differences in weight-related psychological parameters. The main module assignment was computer-based and depended on the results of the Revised Illness Perception Questionnaire (IPQ-R), a standardized questionnaire adapted to overweight and obesity that measures illness beliefs (eg, “my overweight strongly affects the way others see me”) and causal attribution of overweight (eg, “my emotional state, e.g. feeling down, lonely, anxious, empty”) [79]. Participants completed the IPQ-R at the baseline assessment. Each of the 32 items were rated on a 5-point Likert scale ranging from 1 (“strongly disagree”) to 5 (“strongly agree”). In this study, the internal consistency of the scale was good (Cronbach α=.714). Scales were regenerated with higher means representing severe problems on the related psychological parameters associated with overweight and obesity (eg, EE). Of the 5 dimensions, 3 on which the participants reported the highest impairments were tailored to the participants (computer-based tailoring). In addition to the computer-based tailoring feature, individual adaption of content and functions was enabled (self-tailoring). Each module was presented in either a men-specific (*version B*) or a women-specific version (*version A*; “App features” section and Multimedia Appendix 1). The app additionally contained customization features to enhance the adherence to the intervention [80]. In particular, the participants could choose between different coaches at the beginning of the 12-week intervention. A total of 4 different coaches were introduced: 2 men and 2 women coaches depicted as being either more friendly (eg, informal and motivating tips) or more professional (eg, formal and directive tips).

### Measurements

#### Engagement With the App

Use patterns were retrieved from individual app data and subsequently analyzed. Actions were defined as time slots of active engagement with the app, for example, log-in to the app and processing a session (use frequency). Inactivity for 20 minutes defined the completion of one action. The overall app use time was calculated in minutes (use time). The participants who used the app at least 12 times (actions) and for 120 minutes within the 12-week intervention were defined as being compliant with the I-GENDO app.

#### Satisfaction With the App

At the end of the conclusion module, the users could give feedback about their satisfaction with the app and the relevance and daily usefulness of the app on scales ranging from 0 (“not at all”) to 100 (“very much”). In the last session of each module, participants could evaluate how satisfied they were with the corresponding module.

#### Usability Rating of the App

After the 12-week intervention, the mHealth App Usability Questionnaire for stand-alone mHealth apps used by patients was administered [81]. The original English questionnaire was translated into German by a member of the research group and retranslated by a native speaker. Deviations were discussed and subsequently adjusted. The self-report questionnaire consisted of 18 items, which were scored on a scale from 1 (“strongly disagree”) to 7 (“strongly agree”), with higher means reflecting higher usability. Prior research indicated good psychometric properties of the English version of the mHealth App Usability Questionnaire [81]. In this study, the internal consistency of the total scale was excellent (Cronbach α=.935).

### Data Analysis

All analyses were conducted using SPSS for Windows (version 26.0; IBM Corp) and Excel (version 16.0; Microsoft Corp). App data were retrieved from Apache CouchDBTM. Descriptive analyses were conducted using percentages and frequencies for categorical variables and means and SDs for continuous variables. *Chi-square* distributions that compared categorical variables between genders were implemented, and Bonferroni-adjusted independent 2-tailed *t* tests were conducted to compare metrically scaled variables. Mann-Whitney *U* tests were conducted to compare results between genders on nonnormally-distributed variables. Friedman tests and Dunn-Bonferroni post hoc tests were implemented to compare app engagement between genders over the 12 weeks of intervention.

## Results

### Participants

We found no significant gender differences in age, BMI, marital status, and education level at baseline (Table 1).

**Table 1:** Sociodemographic factors (N=116).

Characteristic	Overall	Women (n=77)	Men (n=39)	Women vs men		
				*2-tailed t* test (*df*)	Chi-square (*df*)	*P* value^a^
Age (years), mean (SD)	47.28 (11.66)	46.40 (12.22)	49.00 (10.38)	1.14 (114)	N/A^b^	.26
BMI (kg/m^2^), mean (SD)	33.58 (3.79)	33.75 (3.69)	33.23 (4.02)	0.70 (114)	N/A	.49
Marital status (yes), n (%)^c^	91 (78.4)	57 (74)	34 (87)	N/A	1.9 (1)	.17
Education (university), n (%)^d^	36 (31)	25 (32)	11 (28)	N/A	0.1 (1)	.80

^a^Bonferroni-adjusted *P* values.

^b^N/A: not applicable.

^c^Number of participants in a relationship.

^d^Number of participants with a university degree.

### Tailoring

**Figure 3:**
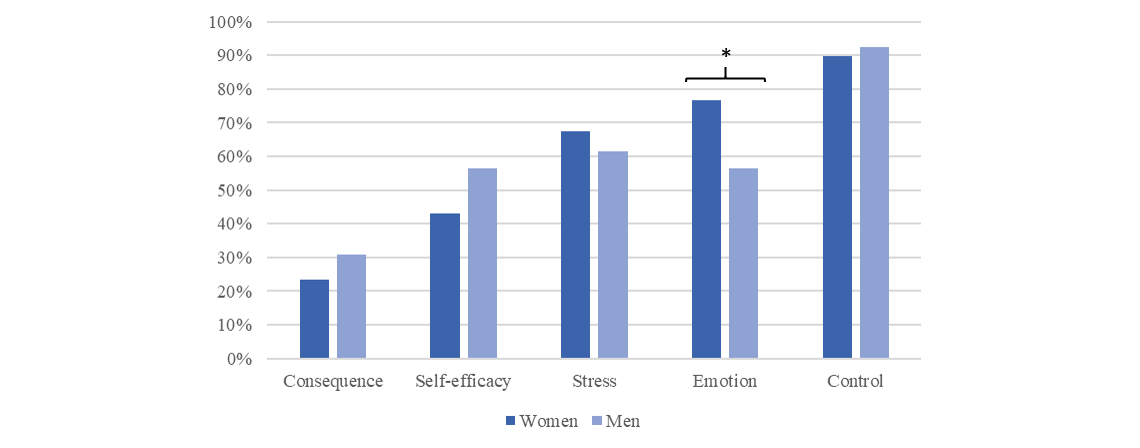
Assigned full-version modules (computer-based tailoring) in percentage (*P<.005).

Three main modules were tailored to each of the 116 participants by computer-based tailoring according to their IPQ-R results (see the section *Tailoring*). Most participants (105/116, 90.5%) received the *control module*, followed by the *emotion module* (81/116, 69.8%), *stress module* (76/116, 65.5%), and *self-efficacy module* (55/116, 47.4%). One-quarter of the participants (30/116, 25.9%) received the *consequence module*. Figure 3 illustrates the module assignments for the participating men and women separately. Significantly more women obtained the *emotion module* than men (*χ*^2^_1_=4.1; *P*=.04; φ=0.21). The genders did not differ in the assignment of the *consequence* (*χ*^2^_1_=0.4; *P*=.53), *self-efficacy* (*χ*^2^_1_=1.6; *P*=.23), *stress* (*χ*^2^_1_=0.2; *P*=.66), or *control module* (*χ*^2^_1_=0.02; *P*=.89).

**Figure 4:**
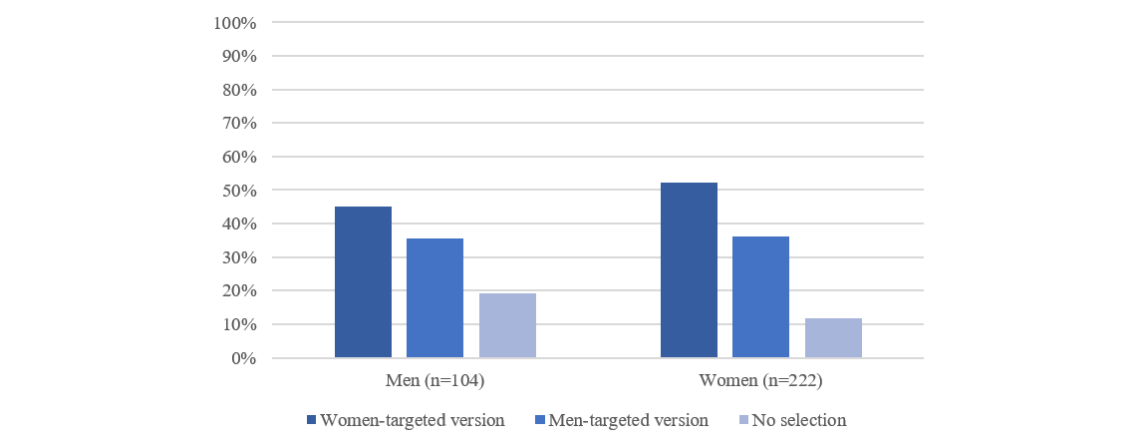
Module version assignments (self-tailoring) in percentage (total choices: N=326).

As described earlier, at the beginning of each module, the participants were instructed to choose between either a women-targeted or a men-targeted version (self-tailoring). In 50% (163/326) of the choices, the women-targeted versions were selected (women: 116/222, 52.3%; men: 47/104, 45.2%). In 35.9% (117/326) of the choices, the men-targeted versions were selected (women: 80/222, 36%; men: 37/104, 35.6%). In the remaining 14.1% (46/326) of the choices, no selection was made (Figure 4). When the participants did choose a version, they chose version A 58.2% (163/280) of the time (women: 116/196, 59.2%; men: 47/84, 56%).

Another customization feature of the intervention was the selection of an accompanying coach when starting the app for the first time. Most women (35/74, 47%) chose a friendly woman coach, 19% (14/74) chose a professional man coach, 18% (13/74) chose a friendly man coach, and 16% (12/74) chose a professional woman coach. Coach assessment in men was more balanced, with 34% (12/35) choosing a friendly woman coach, 23% (8/35) choosing a friendly man or professional woman coach, and 20% (7/35) choosing a professional man coach. No significant gender differences were found in coach assessment (*χ*^2^_3_=1.9; *P*=.60).

### Engagement With the App

Of the 116 participants in the intervention group, 109 actively participated in the app intervention phase. During the 12-week intervention period, the use frequency and use time were recorded.

We found significant gender difference in use frequency (*U*=908.00; *z* score=−2.51; *P*=.01; *r*=−0.24) and use time (*U*=736.00; *z* score=−3.63; *P*<.001; *r*=−0.35). The participating women used the app 97 (SD 88.03) times and for 625 (SD 427.94) minutes on average throughout the intervention, whereas the participating men used the app 56 (SD 45.62) times and for 347 (SD 285.68) minutes on average. In total, 82.6% (90/109) of the users were compliant with the app (women: 63/74, 85%; men: 27/35, 77%).

**Figure 5:**
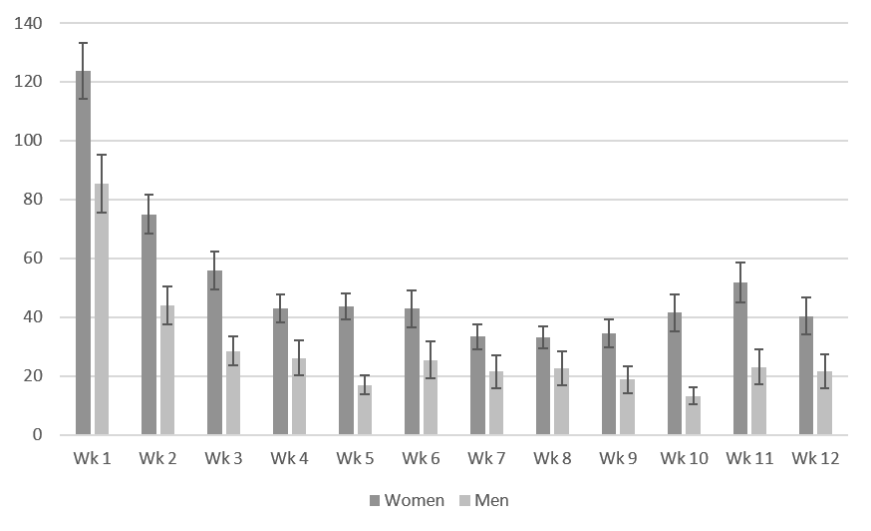
Use time per week in minutes (means and SEs of means).

**Figure 6:**
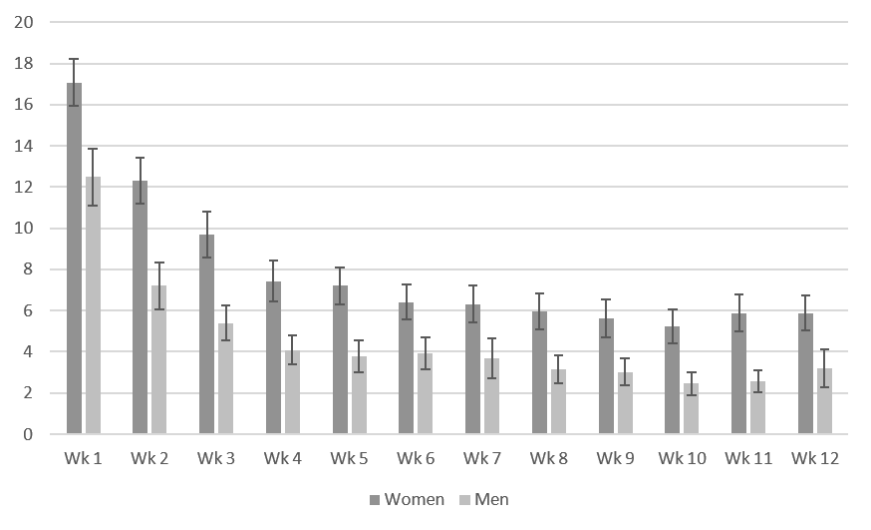
Use frequency per week in actions (means and SEs of means).

During the 12-week intervention phase, the use time (*χ*^2^_11_=126.03; *P*<.001) and use frequency (*χ*²_11_=139.51; *P*<.001) of the participating men (n=35) decreased (Figures 5 and 6). The use time, (*χ*^2^_11_=231.34; *P*<.001) and use frequency (*χ*^2^_11_=309.16; *P*<.001) of the participating women (n=74) also decreased. Dunn-Bonferroni post hoc tests revealed a significant decrease in use time within the first 3 weeks of intervention (*z* score=3.99; *P*<.001; *r*=0.46). From week 3 to week 12, use time and frequency leveled off at approximately 6.56 (SD 7.21) actions per week and 41.99 (SD 34.03) minutes per week for the participating women and 3.53 actions per week (SD 3.36) and 21.75 minutes per week (SD 21.88) for the participating men. We found no gender differences in use time progress (*U*=1075.00; *z* score=−1.43; *P*=.15) and use frequency progress (*U*=1106.00, *z* score=−1.23; *P*=.22) during the 12-week intervention period.

### Evaluation of the App

After completion, 41 participants evaluated the I-GENDO app. On average, the overall satisfaction with the app was high (mean 85.54, SD 19.36). In addition, the relevance of the content (mean 83.34, SD 20.03) and daily life usefulness (mean 78.95, SD 22.24) were evaluated as satisfactory. Of the main modules, the *stress module* (n=36) was rated best (mean 82.92, SD 14.05), followed by the *emotion module* (n=50; mean 81.66, SD 16.45), the *control module* (n=60; mean 80.47, SD 18.08), the *self-efficacy module* (n=29; mean 78.48, SD 17.66), and finally the *consequence module* (n=16; mean 67.75, SD 21.68).

In addition to the evaluation, the usability of the app was assessed using a standardized questionnaire (see the section *Usability Rating of the App*). The usability of the app was rated, on average, with 71% satisfaction (mean 5.00, SD 1.08 points; maximum: 7.00 points). No gender differences could be found between the usability ratings of men (mean 4.72, SD 1.07) and women (mean 5.13, SD 1.07; t_99_=−1.76; *P*=.08).

## Discussion

### Overview

We aimed to introduce the I-GENDO app, a tailored gender-sensitive mHealth weight loss intervention, and present results from its process evaluation data. Therefore, data from the intervention arm of the I-GENDO project were analyzed. The sample included 116 (n=77, 66.4% women) individuals with overweight and obesity.

### Principal Findings

We developed a module-based 12-week intervention combining computer-based and self-tailoring features. Most participants (105/116, 90.5%) received the *control module*, which focused on self-regulation skills of food craving. The *stress module* was assigned to 65.5% (76/116) of the participants, and the *self-efficacy module* to 47.4% (55/116). The *consequence module* was obtained by 25.9% (30/116) of the participants*.* Significantly more women (59/77, 77%) than men (22/39, 56%) received the *emotion module*. Another tool of the intervention was the implementation of gender-sensitive self-tailoring features. We developed women- and men-targeted versions of the main modules. At the beginning of each module, participants could choose between the 2 versions. Among the participants who chose a version, *version A* was chosen 58.2% (163/280) of the time (women: 116/196, 59.2%; men: 47/84, 56%), which means that among both genders, the women-targeted module versions were predominantly selected.

In total, 82.6% (90/109) of the participants (women: 63/74, 85%; men: 27/35, 77%) were compliant with the I-GENDO app during the intervention phase. Use time and frequency significantly decreased during the 12-week intervention phase for both genders. After the first 3 weeks of intervention, use time leveled off and remained stable at approximately 42 minutes per week for the participating women and 22 minutes per week for the participating men. Similarly, use frequencies were approximately stable as of week 3 for both genders. Compared with the women, the men used the app infrequently and spent less time with the app. Nevertheless, the average use times and frequencies in both genders were satisfactory even in the last weeks.

The overall satisfaction with the app was high, with almost 86% (86/100) approval. In addition, the daily life usefulness and relevance of the content were ranked satisfactory by 79% (79/100) and 83% (83/100) of participants, respectively. The highest-rated main module was the *stress module* (83/100, 83%), but even the satisfaction with the *consequence module* was acceptable (68/100, 68%). In general, the usability ratings indicated that the I-GENDO intervention was good, averaging 5.0 out of 7.0 points (71%).

### Comparison With Prior Work

The heterogeneous computer-based administration of the main modules supports the tailoring feature. The *control module* was assigned to most participants. This is in line with the observation that decreased food-related inhibitory control is regularly associated with overweight and obesity [49,82,83]. Gender differences were found in the computer-based assignments of the *emotion module*, which significantly more women obtained. The module focused on dysfunctional emotion regulation and associations between negative emotions and (eating) behavior. EE refers to problems in the distinction between physiological appetite and eating as a strategy to cope with negative feelings [84]. EE is correlated with higher weight, severe depression symptoms, and the consumption of sweet energy-dense foods [85]. More women report negative emotions as causes for their overweight and engage more often in EE compared with men [50,85,86]. EE is associated with less intuitive eating by women, which could be a barrier to the implementation of healthy eating behaviors [87]. Studies indicate that more women undergo weight loss treatment, whereas participating men lose more absolute weight [29]. Focusing more on EE in treatment might contribute to a close in this gap. In addition, previous studies indicated that a relevant subgroup of individuals with overweight and obesity exhibit addiction-like eating behavior (ie, food addiction [FA]), characterized by an impaired food-related inhibitory control, EE, and food craving [88,89]. The prevalence of FA is higher in women than in men and is among other factors associated with higher BMI, dysfunctional eating behavior, and psychological distress [90,91]. Some studies reported lower adherence to and decreased effectiveness of WLPs in individuals experiencing FA, whereas others found no influence of FA on the success of WLPs [92-95]. As the *control* and *emotion* modules implement the treatment of dysfunctional EE behavior and exercises to improve food-related inhibitory control, participants experiencing FA might especially benefit from the intervention. Thus, the association between FA and the effectiveness of our intervention should be further investigated.

One-quarter of the participants received the *consequences module*, which focused on weight-related discrimination and the improvement of self-esteem and body image, as well as the social competences to deal with discrimination. The extent of this use might explain the prevalence of weight discrimination being higher in our sample than in the results of a representative German study reporting prevalence rates ranging from 5.6% to 18.7% in individuals with overweight and obesity (classes I and II) [96]. We hypothesized that individuals who have experienced discrimination might prefer seeking WLPs based on psychological rather than lifestyle features. Moreover, in our study, the *consequence module* was assigned to more men (12/39, 31%) than women (18/77, 23%), which appears to be in contrast to the results of the previously cited study that reported double the prevalence of weight-based discrimination in women [96]. The anonymity of a smartphone-based intervention combined with the opportunity to receive specialized psychological support targeted to individual needs could have been particularly appealing for men who had experienced weight-related discrimination and were affected by the consequences of their overweight. Nevertheless, the module generally focused on weight-related emotional and physical consequences, which might be appealing to individuals with overweight and obesity regardless of whether they experienced discrimination.

Gender differences in health care services are an important consideration for the improvement of treatment outcomes [97]. Prior studies have indicated gender differences in eating behavior, as well as the psychological factors associated with weight gain and maintenance, highlighting the need for gender-targeted weight loss interventions [29,40]. As the effectiveness of gender dichotomous tailoring does not significantly differ from that of gender-neutral interventions [45], we implemented gender-sensitive self-tailoring features. The participants could choose between 2 gender-targeted versions at the beginning of the modules. The selection of the versions was heterogeneous, with most participants choosing women-targeted versions. This result supports the idea of gender-sensitive interventions to overcome gender binary [46]. However, its influence on the effectiveness of the intervention needs to be further investigated.

In complex digital interventions, the consideration of relevant process evaluation data (eg, usability testing and use patterns) is crucial before interpreting the effectiveness of the intervention [98]. The compliance with the app was satisfactory (90/109, 82.6%) and comparable with other studies. Signal et al [99] developed an eHealth intervention for prediabetes and diabetes self-management. They reported that 74% of the participants were actively engaged (ie, any use data were detected at any time throughout the 16-week intervention). Ruf et al [100] developed an mHealth intervention that assesses event-contingent dietary intake and physical activity, as well as relevant psychological parameters. Compliance, defined as the percentage of complete prompts within the total number of prompts received, was 80%. Another mHealth intervention focused on the management of food-related impulses to facilitate weight loss [101]. In that study, the completion rate (the number of participants who provided data at the 3-month follow-up) was 76%. These findings suggest that our compliance rate is comparable or even higher, although the differences in operational definitions cloud the interpretation.

Throughout the intervention, the use time and frequency decreased in both genders. Decreases in engagement were also reported in other studies; that is, in those with extended intervention periods [99,102]. Reductions in engagement and high dropouts are typical for internet-based interventions and are caused by a variety of reasons [103]. We hypothesized that the reduction in engagement observed in our study might be associated with the high number of competing commercial digital weight loss interventions, which might be less demanding, compared with psychological interventions. Moreover, the intervention phase of our study fell within the first and second lockdowns of the COVID-19 pandemic in Germany in 2020. During this period, the level of psychological distress increased, and vulnerable people engaged more often in dysfunctional eating patterns (ie, EE) [104]. In addition, many people were affected by short-term work or job losses and subsequent income losses [105]. It is likely that people neglected the intervention during this burdensome period.

The results from previous studies on the adherence to mHealth interventions are heterogeneous, with some reporting higher engagement in men [29,40,106] and others reporting higher engagement in women [99]. In our study, women used the app more frequently and spent more time on it. In the general German population, women report higher smartphone use time (mean 167 min/day) than men (mean 154 min/day), which might at least partially explain these differences [107]. Moreover, women are more interested in body appearance and health-related topics than men and use the internet more frequently for medical and health research [108-110]. Studies have also reported that women are more likely to use mHealth interventions focusing on nutrition and self-care apps, whereas men are more likely to use fitness apps [111-113]. Therefore, the lower engagement of the participating men in this study might be because the app focused on psychological rather than physiological determinants of overweight and obesity.

As reported in a recently published systematic review [114], other studies on mHealth interventions have either failed to report gender differences in the adherence to and usability of these interventions or reported results from biased samples with approximately 90% of women [115-117]. Given that higher engagement in mHealth interventions is usually associated with better outcomes [22,24,118], we propose that the samples in future studies should be more balanced with regard to gender and implement gender-sensitive feasibility and usability testing. Overall, the compliance with the app (90/109, 82.6%) and satisfaction with the app (86/100, 86%) were high and comparable with those of other mHealth interventions [99-101,119]. The usability of the app was rated with 71% (5.0/7.0 points) satisfaction. Other evidence-based mHealth weight loss interventions reported comparable or even lower usability scores, between 61.9% and 69.3% [100,119]. In addition, Ferrara et al [120] reviewed the usability of commercial weight loss apps, which can be downloaded from Google Play and the Apple Store. Scientists ranked the usability of these apps between 47% and 89%.

### Limitations

In our study, men and women differed in the assignment of main modules, which focused on psychological parameters associated with the development and maintenance of overweight and obesity. Interestingly, most men and women selected the women-targeted versions of the main modules. Given that the participants were blind to the gender-targeted manipulation, we suggest that the selections were not influenced by social desirability. Future studies should distinguish between gender differences based on the results from explicit and implicit assessments to adjust for social norms. Moreover, the participants were forced to select one version at the beginning of each module and were not allowed to switch versions. A reasonable approach could be to allow participants to test both versions to enhance their adherence to the app. In addition, it should be verified whether the introductions of the versions sufficiently hint at different module content.

It should be noted that only few participants (41/109, 37.6%) evaluated the app after completion. The evaluation was voluntary and was assessed at the end of the last session of the intervention. Therefore, results regarding satisfaction with the app and the main modules should be interpreted cautiously.

The results from the process evaluation revealed that men and women differed in their app use. Women used the app more frequently and longer than men. Most of the scientists involved in the development process were women. Therefore, the women-targeted features of the app might have been more salient and thus confounded the selection by both genders. This methodological aspect might subsequently explain the higher use patterns of the participating women. Future studies or revisions of the app intervention should involve men scientists.

### Conclusions

In summary, given the high diversity in module assignment, we hypothesize that tailoring was successfully implemented in the intervention. The heterogeneous selection of the gender-targeted features might underscore the need for gender-sensitive (self-tailoring and blind choice) instead of gender dichotomous (computer-based tailoring) targeting but could also hint at methodological limitations, which need to be considered and further investigated in future studies. Further studies need to clarify whether the reported gender differences in the use and evaluation of the app confound the effectiveness and sustainability of the I-GENDO intervention.

## Appendix

Multimedia Appendix 1 Evidence, content, and adaptions of the gender-sensitive main modules.

## Abbreviations

EE: emotional eating
FA: food addiction
IPQ-R: revised illness perception questionnaire
mHealth: mobile health
WLP: weight loss program
