# Supplementary material for: A Tailored Gender-Sensitive mHealth Weight Loss Intervention (I-GENDO): Development and Process Evaluation
Source: JMIR Form Res. 2022 Oct 27;6(10):e38480. doi: 10.2196/38480 (PMC9650578; doi:10.2196/38480)
Supplement: Multimedia Appendix 1 [file formative_v6i10e38480_app1.docx]

Supplementary Material 1: Evidence, content and adaptions of the gender-sensitive main modules

| Evidence [origin] | Module | Module Content | Behavior Change Techniques[1] | Gender-Specific Adaptations |
| --- | --- | --- | --- | --- |
| - Association between chronic stress (high levels of glucocorticoids) and unfavourable eating styles, negative affect and body weight [2] - Stress is a risk factor for obesity [3] - Women and men with obesity report more stressful live events than normal weight individuals [4] - Assumption that men with obesity tend to prefer individual, self-guided programs, which focus on facts rather than “feelings”, prefer factual information on how to lose weight, men tend to prefer technical information and becoming an expert of their condition [5]–[7] | *Stress Module* | - psychoeducation: general information about stress development, responses and management - exercises to identify individual stress and stress responses and (dys)functional coping behavior - exercises to identify associations between stress and individual eating behavior, weight gain and well-being [8], [9] - exercises to improve personal stress management and coping skills based on the stress management training of Gerd Kaluza (i.e., time management, mindfulness, problem solving)[10], [11]. | Self-monitoring of behavior  Social support (emotional)  Behavior substitution  Behavioral rehearsal/practice  Action planning  Problem solving | **Version A:**   - psychoeducation based on relatable case examples   **Version B:**   - psychoeducation based on relatable gender-specific statistics, facts and data |
| - Association between emotional eating and weight gain [12] - Women with obesity report a high burden by emotional states [focus groups]* - Gender differences in emotional competences, emotional eating, usage of emotion-regulation strategies , women tend to engage in rumination more often than men [13], [14] - Women tend to be more aware of their emotions and engage in more (mal)adaptive emotion regulation strategies than men [14] | *Emotion Module* | - psychoeducation: general information about emotions and development of emotions (emergence of emotions due to weight and weight discrimination) and associations to behavior - exercises to identify associations between emotions and (eating) behavior[15] - exercises to build functional emotion regulation skills based on the Dialectical behavior therapy (DBT)[9], [16] | Self-assessment of affective consequences  Self-monitoring of behavior  Behavior substitution  Behavioral experiments  Emotional consequences  Regulate negative emotions | **Version A:**   - associations between cognition and dysfunctional beliefs (ABC model) [17] - focusing on rumination as dysfunctional emotion regulation strategy   **Version B:**   - identifying, labelling emotions, building up basic emotional competences (DBT manual) [16] - focusing on suppression as a dysfunctional emotion regulation strategy |
| - Men and women with overweight and obesity experience weight discrimination in multiple areas of life, gender difference in consequences and coping behavior [18], [19] - Men with obesity experience weight-loss as a competition and a project against themselves, commitment to “masculine” hard work [focus groups]* [20] - Men reported more physical and women reported more emotional consequences of overweight [focus groups]* | *Consequences Module* | - psychoeducation: general information about weight-related consequences (e.g., weight discrimination) [9] - associations between weight discrimination and weight management - exercises to improve self-esteem and body image[8], [15] - exercises to improve social competences (self-confident behavior) based on social competence group trainings [21] | Emotional consequences  Social and environmental consequences  Self-monitoring of behavior  Social support (general)  Behavioral experiments | **Version A:**   - highlighting the emotional consequences of (internalized) weight discrimination - CBT-based tools to improve body image and social competences [9], [21]   **Version B:**   - highlighting the physical consequences of overweight - offering competitive challenges and tasks to improve physical well-being and social competences |
| - Association between weight, eating behavior and impulsivity [22], [23] - Men with obesity tend to prefer weight-loss programs which enable control and autonomy and promote problem-solving; loss of control is incoherent with male role [6], [7], [24] - Women with obesity reported higher levels of restraint eating, rigid avoidance of situations and food cues that trigger loss of control and a dichotomous thinking style [focus groups]* - Lack of long-term regulation strategies [focus groups]* | *Control Module* | - psychoeducation: general information about the association between self-regulation and impulsivity and weight-related outcomes [8], [9], [15] - identify associations between loss of control eating and contextual or personal aspects - exercises to improve self-regulation in general and food-related inhibitory control in particular in individually meaningful critical situations [25] | Behavior substitution  Behavioral rehearsal/practice  Generalization of a target behavior  Prompts/cues  Self-monitoring of behavior  Avoiding/changing exposure to cues for the behavior  Behavioral experiments | **Version A:**   - psychoeducation based on relatable case examples - differentiating eating types (e.g., restrictive eating) - CBT-based strategies to improve food-related inhibition (behavioral analysis)[8], [25]   **Version B:**   - psychoeducation based on relatable gender-specific statistics, facts, data (e.g., dual process theory)[26] - Go/No-Go training (gamification) tailored to individual attractive high-caloric food to improve food-related inhibition [27] |
| - Self-efficacy is positively associated with weight management [28], [29] - Women and men that experienced successful long-term weight loss maintenance reported higher levels of self-efficacy [focus groups]* - Men with obesity experience weight-loss as a competition and a project against themselves, commitment to “masculine” hard work [focus groups]* +[20] | *Self-efficacy Module* | - psychoeducation: general information about eating and movement-specific self-efficacy - association between self-efficacy, goal-setting and weight management[8] - exercises to improve weight management-related self-efficacy based on Bandura’s 4 sources of efficacy beliefs [30] | Self-monitoring of behavior  Goal setting  Review behavior goal(s)  Social support (general)  Modeling of the behavior  Social comparison  Focus on past success  Verbal persuasion to boost self-efficacy | **Version A:**   - focussing on improving mastery experiences (internal attribution) and social support - CBT-based strategies to improve sources of efficacy beliefs   **Version B:**   - Approach-Avoidance Training (gamification) to improve food-related self-efficacy and offering competitive challenges to implement changes in everyday life |

^*^ Results of qualitative content analyses based on focus groups with women (*n* = 18) and men (*n* = 12) with overweight or obesity.

References

[1] S. Michie *et al.*, “The behavior change technique taxonomy (v1) of 93 hierarchically clustered techniques: building an international consensus for the reporting of behavior change interventions,” *Ann. Behav. Med.*, vol. 46, no. 1, pp. 81–95, 2013, doi: 10.1007/s12160-013-9486-6Zhou.

[2] M. F. Dallman *et al.*, “Chronic stress and obesity: A new view of ‘comfort food,’” *Proc. Natl. Acad. Sci. U. S. A.*, vol. 100, no. 20, pp. 11696–11701, Sep. 2003, doi: 10.1073/pnas.1934666100.

[3] R. Sinha and A. M. Jastreboff, “Stress as a Common Risk Factor for Obesity and Addiction,” *Biol. Psychiatry*, vol. 73, no. 9, pp. 827–835, May 2013, doi: 10.1016/j.biopsych.2013.01.032.

[4] D. Barry and N. Petry, “Gender differences in associations between stressful life events and body mass index,” *Prev. Med.*, vol. 47, no. 5, pp. 498–503, Nov. 2008, doi: 10.1016/j.ypmed.2008.08.006.

[5] E. Bennett and B. Gough, “In pursuit of leanness: the management of appearance, affect and masculinities within a men’s weight loss forum,” *Health Lond. Engl. 1997*, vol. 17, no. 3, pp. 284–299, May 2013, doi: 10.1177/1363459312454149.

[6] P. Galdas *et al.*, “The accessibility and acceptability of self-management support interventions for men with long term conditions: a systematic review and meta-synthesis of qualitative studies,” *BMC Public Health*, vol. 14, p. 1230, Nov. 2014, doi: 10.1186/1471-2458-14-1230.

[7] C. Robertson *et al.*, “Systematic reviews of and integrated report on the quantitative, qualitative and economic evidence base for the management of obesity in men,” *Health Technol. Assess. Winch. Engl.*, vol. 18, no. 35, pp. v–vi, xxiii–xxix, 1–424, May 2014, doi: 10.3310/hta18350.

[8] S. Becker, S. Zipfel, and M. Teufel, *Psychotherapie der Adipositas: Interdisziplinäre Diagnostik und differenzielle Therapie*. Kohlhammer Verlag, 2015.

[9] Z. Cooper and C. G. Fairburn, “A new cognitive behavioural approach to the treatment of obesity,” *Behav. Res. Ther.*, vol. 39, no. 5, pp. 499–511, 2001.

[10] G. Kaluza, *Stressbewältigung: Trainingsmanual zur psychologischen Gesundheitsförderung*. Springer-Verlag, 2018.

[11] G. Kaluza, “Gelassen und sicher im Stress-Das Stresskompetenz-Buch,” *Springer Heidelb.*.

[12] J. Bennett, G. Greene, and D. Schwartz-Barcott, “Perceptions of emotional eating behavior. A qualitative study of college students,” *Appetite*, vol. 60, no. 1, pp. 187–192, Jan. 2013, doi: 10.1016/j.appet.2012.09.023.

[13] J. K. Larsen, T. van Strien, R. Eisinga, and R. C. M. E. Engels, “Gender differences in the association between alexithymia and emotional eating in obese individuals,” *J. Psychosom. Res.*, vol. 60, no. 3, pp. 237–243, Mar. 2006, doi: 10.1016/j.jpsychores.2005.07.006.

[14] S. Nolen-Hoeksema and A. Aldao, “Gender and age differences in emotion regulation strategies and their relationship to depressive symptoms,” *Personal. Individ. Differ.*, vol. 51, no. 6, pp. 704–708, Oct. 2011, doi: 10.1016/j.paid.2011.06.012.

[15] S. Munsch and A. Hilbert, *Übergewicht und Adipositas*, vol. 59. Hogrefe Verlag, 2015.

[16] M. Bohus and M. Wolf-Arehult, *Interaktives Skillstraining für Borderline-Patienten*. Schattauer Verlag, 2013.

[17] G. Eismann and C.-H. Lammers, *Therapie-Tools Emotionsregulation: Mit E-Book inside und Arbeitsmaterial*. Beltz, 2017.

[18] R. M. Puhl and K. D. Brownell, “Confronting and coping with weight stigma: an investigation of overweight and obese adults,” *Obes. Silver Spring Md*, vol. 14, no. 10, pp. 1802–1815, Oct. 2006, doi: 10.1038/oby.2006.208.

[19] R. M. Puhl and C. A. Heuer, “The Stigma of Obesity: A Review and Update,” *Obesity*, vol. 17, no. 5, pp. 941–964, 2009, doi: https://doi.org/10.1038/oby.2008.636.

[20] D. Couch, G.-S. Han, P. Robinson, and P. Komesaroff, “Men’s weight loss stories: How personal confession, responsibility and transformation work as social control,” *Health (N. Y.)*, vol. 23, no. 1, pp. 76–96, Jan. 2019, doi: 10.1177/1363459317724855.

[21] R. Hinsch and U. Pfingsten, *Gruppentraining sozialer Kompetenzen: GSK; Grundlagen, Durchführung, Anwendungsbeispiele*. Beltz, 2015.

[22] S. Bartholdy, B. Dalton, O. G. O’Daly, I. C. Campbell, and U. Schmidt, “A systematic review of the relationship between eating, weight and inhibitory control using the stop signal task,” *Neurosci. Biobehav. Rev.*, vol. 64, pp. 35–62, May 2016, doi: 10.1016/j.neubiorev.2016.02.010.

[23] K. Houben, C. Nederkoorn, and A. Jansen, “Eating on impulse: the relation between overweight and food-specific inhibitory control,” *Obes. Silver Spring Md*, vol. 22, no. 5, pp. E6-8, May 2014, doi: 10.1002/oby.20670.

[24] J. B. Carey, K. K. Saules, and M. M. Carr, “A qualitative analysis of men’s experiences of binge eating,” *Appetite*, vol. 116, pp. 184–195, Sep. 2017, doi: 10.1016/j.appet.2017.04.030.

[25] H. Preuss, K. Schnicker, and T. Legenbauer, *ImpulsE zur Verbesserung der Impuls-und Emotionsregulation: Ein kognitiv-verhaltenstherapeutisches Behandlungsprogramm*. Hogrefe Verlag, 2018.

[26] W. Hofmann, M. Friese, and F. Strack, “Impulse and self-control from a dual-systems perspective,” *Perspect. Psychol. Sci.*, vol. 4, no. 2, pp. 162–176, 2009, doi: 10.1111/j.1745-6924.2009.0116.x.

[27] L. Lavagnino, D. Arnone, B. Cao, J. C. Soares, and S. Selvaraj, “Inhibitory control in obesity and binge eating disorder: A systematic review and meta-analysis of neurocognitive and neuroimaging studies,” *Neurosci. Biobehav. Rev.*, vol. 68, pp. 714–726, 2016.

[28] M. M. Clark, D. B. Abrams, R. S. Niaura, C. A. Eaton, and J. S. Rossi, “Self-efficacy in weight management,” *J. Consult. Clin. Psychol.*, vol. 59, no. 5, pp. 739–744, 1991, doi: 10.1037/0022-006X.59.5.739.

[29] R. M. Richman, G. T. Loughnan, A. M. Droulers, K. S. Steinbeck, and I. D. Caterson, “Self-efficacy in relation to eating behaviour among obese and non-obese women,” *Int. J. Obes. Relat. Metab. Disord. J. Int. Assoc. Study Obes.*, vol. 25, no. 6, pp. 907–913, Jun. 2001, doi: 10.1038/sj.ijo.0801606.

[30] J. A. Linde, A. J. Rothman, A. S. Baldwin, and R. W. Jeffery, “The impact of self-efficacy on behavior change and weight change among overweight participants in a weight loss trial.,” *Health Psychol.*, vol. 25, no. 3, p. 282, 2006.
